# Dietary Inflammatory Index during Pregnancy and the Risk of Intrapartum Fetal Asphyxia: The Japan Environment and Children’s Study

**DOI:** 10.3390/nu12113482

**Published:** 2020-11-13

**Authors:** Hyo Kyozuka, Tsuyoshi Murata, Toma Fukuda, Akiko Yamaguchi, Aya Kanno, Shun Yasuda, Akiko Sato, Yuka Ogata, Masahito Kuse, Mitsuaki Hosoya, Seiji Yasumura, Koichi Hashimoto, Hidekazu Nishigori, Keiya Fujimori, the Japan Environment and Children’s Study (JECS) Group

**Affiliations:** 1 Fukushima Regional Center for the Japan Environmental and Children’s Study, 1 Hikarigaoka, Fukushima 960-1295, Japan; tuyoshim@fmu.ac.jp (T.M.); t323@fmu.ac.jp (T.F.); akiko-y@fmu.ac.jp (A.Y.); ayao0827@fmu.ac.jp (A.K.); room335@fmu.ac.jp (S.Y.); asato@fmu.ac.jp (A.S.); yuka-o@fmu.ac.jp (Y.O.); kuse2018@fmu.ac.jp (M.K.); mhosoya@fmu.ac.jp (M.H.); yasumura@fmu.ac.jp (S.Y.); don@fmu.ac.jp (K.H.); nishigo@fmu.ac.jp (H.N.); fujimori@fmu.ac.jp (K.F.); 2 Department of Obstetrics and Gynecology, Fukushima Medical University School of Medicine, 1 Hikarigaoka, Fukushima 960-1295, Japan; 3 Department of Pediatrics, Fukushima Medical University School of Medicine, 1 Hikarigaoka, Fukushima 960-1295, Japan; 4 Department of Public Health, Fukushima Medical University School of Medicine, 1 Hikarigaoka, Fukushima 960-1295, Japan; 5 Fukushima Medical Center for Children and Women, Fukushima Medical University, 1 Hikarigaoka, Fukushima 960-1295, Japan

**Keywords:** pro-inflammatory diet, pregnancy, dietary inflammatory index, fetal acidosis, umbilical artery pH

## Abstract

We aimed to examine the impact of a daily pro-inflammatory diet during pregnancy on intrapartum fetal acidemia using a large birth cohort study in Japan. We used data on singleton pregnancies in the Japan Environment and Children’s Study (JECS) involving births from 2011 to 2014 through vaginal delivery to calculate the maternal dietary inflammatory index (DII). Participants were categorized according to DII quintiles. A multiple logistic regression model was used to estimate the risk of a pro-inflammatory diet on fetal umbilical artery pH. In total, 56,490 participants were eligible for this study. Multiple regression analysis showed that nulliparous women who had undergone vaginal delivery and were consuming a pro-inflammatory diet had an increased risk of pH < 7.10 (adjusted odds ratio [aOR]: 1.64, 95% confidence interval [CI]: 1.12–2.39). Among these women, the risk of pH < 7.10 was not affected by the duration of labor (aOR: 1.64, 95% CI: 1.11–2.42). In conclusion, following a pro-inflammatory diet during pregnancy is a risk factor for fetal acidosis among nulliparous women undergoing vaginal delivery. A high DII diet during pregnancy may modify the intrapartum fetal heart rate pattern via intrauterine inflammation.

## 1. Introduction

Recently, the concept of pro-inflammatory and anti-inflammatory diets has been reported. Diets that contain artificial trans fats, vegetable oil, refined carbohydrates, or processed meat causing inflammation are referred to as pro-inflammatory diets [1,2,3,4]. In contrast, diets that contain fruits such as berries or cherries, fatty fish, green tea, and extra virgin olive oil reduce inflammation and are termed anti-inflammatory diets [5,6,7,8,9]. The dietary inflammatory index (DII) is used to assess the anti- or pro-inflammatory potential of an individual’s diet [10]. The greater the DII score, the more pro-inflammatory the diet. A high DII has been proved to be associated with non-communicable diseases such as cancer, cardiovascular disease, obesity, type 2 diabetes mellitus, and asthma [11]. However, few studies have conducted about the correlation between high DII during pregnancy and intergeneration effect, reporting associated with fetal growth [12,13].

Fetal acidosis at birth is a major cause of short- and long-term childhood morbidity and mortality, including persistent pulmonary hypertension, hypoxic ischemic encephalopathy, and neurodevelopmental delay [14,15,16]. Umbilical cord pH at birth is frequently used to detect fetal acidosis. It results mainly from the interruption of placental blood flow and subsequent severe fetal hypoxia during labor [17,18]. Intrapartum fetal oxygenation, especially during intrauterine inflammation, is essential for neonatal prognosis because a fetus with inflammation is vulnerable to hypoxic stress, which can result in brain damage [19].

In our previous study using the largest Japanese birth cohort study, we reported that a high DII score (pro-inflammatory effect) before pregnancy associated with leukocytosis during the first trimester increases the risk of preterm birth (PTB) and low birth weight (LBW) [20]. This previous study indicated that a high DII diet during pregnancy also increases the risk of intrauterine inflammation and might affect short-term outcomes such as perinatal asphyxia, especially among women whose first births were by vaginal delivery, which has the potential risk of intrapartum hypoxic stress to the fetus.

This study was conducted to examine the hypothesis that a pro-inflammatory diet during pregnancy is a risk factor for fetal acidosis at birth. Specifically, we asked two questions in this study: (ⅰ) Is fetal acidemia (determined by fetal arterial umbilical cord pH at birth) affected by pro-inflammatory diet during pregnancy? (ⅱ) If so, does parity, which could affect fetal oxygenation at birth, modify the risk of fetal acidosis?

## 2. Materials and Methods

### 2.1. Study Design

In our analysis, we used data from the Japan Environmental Children’s Study (JECS), a government-funded birth cohort study that started in January 2011. The JECS investigated the effect of several environmental factors during pregnancy on children’s health [21]. Participants’ (mothers) eligibility requirements for JECS were as follows: (1) They lived in the Study Area at the time of application and are expected to live in Japan in future; (2) the expected delivery date was between 1 August 2011 and mid-2014; and (3) they could participate in this study without difficulty in understanding or writing Japanese.

The JECS protocol was reviewed and approved by the Ministry of the Environment’s Institutional Review Board on Epidemiological Studies and the ethics committees of all participating institutions. The JECS was conducted in accordance with the Helsinki Declaration, and written informed consent was obtained from all participating women.

### 2.2. Data Collection

The dataset released in June 2016 (dataset: jecs-ag-20160424) was used for this study. This dataset comprised three keys sets of information: (1) a self-reported questionnaire obtained around the participants’ first visit, including medical background (e.g., maternal age, history of previous pregnancy, maternal height, smoking status, and maternal weight before pregnancy), (2) food frequency questionnaire (FFQ), which is a self-reported questionnaire collected during the second/third trimester, and (3) obstetrics outcome that was retrieved from the medical records of the institutions of the participants. In the present analysis, we used the FFQ data that were completed during the second/third trimester. This FFQ used in this study has been previously validated using 12-day weighed food records in adults aged 40–74 years in Japan [22]. For this study, we excluded cases with insufficient data, multifetal gestation, and cases with cesarean delivery. We also excluded cases with obstetric complications, such as preterm birth (PTB) < 37 weeks, low birth weight infant (LBW) < 2500 g, hypertension disorders of pregnancy (HDP), and gestational diabetes mellitus (GDM), as these complications potentially affect fetal oxygenation.

### 2.3. Calculation of DII

The DII score is a comprehensive indicator of daily inflammatory and anti-inflammatory meal contents developed by Shivappa et al. [10]. The greater the positive DII score, the more the pro-inflammatory effect of the diet. On the other hand, a higher negative value indicated a more anti-inflammatory diet. The method of calculation of DII in JECS data has been previously reported [20]. In brief, 30 food parameters were obtained from each participant’s FFQ, including alcohol; β-carotene; vitamins A, B-12, B-6, C, D, and E; carbohydrate; energy; cholesterol; protein; total fat; fiber; saturated fat; monounsaturated fatty acids (MUFAs); polyunsaturated fatty acids (PUFAs); fatty acids (*n*-3 and *n*-6 FAs); niacin; thiamin; riboflavin; iron; magnesium; zinc; selenium; folic acid; garlic; ginger; and onion [20]. The DII of each participant was calculated as follows: First, dietary data were linked to a worldwide database that provided a robust estimate of the mean and standard deviation (SD) for each parameter included in the DII [10]. The *Z* score was calculated by subtracting the standard global average from the reported amount and dividing the results by SD. As the *Z* scores were not normally distributed (right skewing), the *Z* score for each value changed to a centralized percentile score. Then, the percentile centered on the food parameter was multiplied by each food parameter effect score (obtained by reviewing 1943 research articles to determine the relationship between food parameter and inflammation) to obtain a food parameter-specific DII score. Finally, all of it was summed to create an overall DII score for each participant as follow: DII = I1 × P1 + I2 × P2 + … + I30 × P30 (where “I” is the effects of inflammation from reviewed research articles on food parameter effect scores, and “P” is food-specific centered percentile score derived from food data. The minimum/maximum DIIs in pregnant populations one year before pregnancy in previous JECS study were reported to range from −6.16 to +5.80 [20].

### 2.4. Obstetric Outcomes and Confounding Factors

Fetal arterial blood was obtained at the site of delivery, and UmA-pH was measured immediately after delivery. Fetal acidosis was defined as UmA-pH < 7.20, < 7.10, or < 7.00 according to one previous study showing that an UmA-pH threshold of 7.20 is associated with an increased risk of adverse short-term outcomes [23]. UmA-pH threshold of 7.10 is associated with an increased risk of adverse neurological sequelae [18]. Cerebral palsy is thought to occur more frequently at UmA-pH < 7.00 [24]. PTB was defined as delivery before 37 gestational weeks. LBW was defined as birth weight < 2500 g. Pregnant woman with HDP and/or GDM were diagnosed by obstetricians at each institution. Mode of delivery was categorized as vaginal or cesarean delivery. Parity was categorized as nulliparous or multiparous. The following were considered as confounding factors: maternal age, maternal body mass index (BMI), maternal smoking status, and duration of labor. Based on the previous JECS study where maternal age < 20 and ≥ 30 years was reported to be a risk factor for poor obstetrics outcome [25,26], maternal age was categorized into the following three groups: ≤19, 20–29, and ≥30 years. Maternal pre-pregnancy BMI was calculated. Following this, we categorized participants into three BMI groups as follows: <18.5, 18.5–25.0, and ≥25.0 kg/m^2^. A self-reported questionnaire during first trimester provided information on their smoking status during the first trimester: “never smoked,” “quit smoking before pregnancy,” “quit smoking during early pregnancy,” and “kept smoking during pregnancy.” Women who “kept smoking during pregnancy” were considered smokers (smoking category); otherwise, they were considered as non-smokers (non-smoking). Duration of labor was defined as length (hour) from uterine contraction every ten minutes to the end of their delivery.

### 2.5. Statistical Analyses

Participants were categorized according to quintiles (Q1 was the most anti-inflammatory group, whereas Q5 was the most pro-inflammatory group). First, maternal characteristics and obstetrics outcomes were summarized according to DII category. Then, because UmA-pH at delivery is strongly affected by the fetal oxidative condition before delivery and especially by parity [27], the participants were also stratified into two groups on the basis of parity (nulliparous or multiparous), and DII and obstetrics outcomes were compared between the stratified groups. One-way ANOVA, T-tests, Mann–Whitney U tests were conducted to compare continuous variables, and the Chi-square test was used to compare categorical variables, as appropriate. After stratification by parity, logistic regression models were used to calculate the adjusted odds ratios (aORs) and 95% confidence intervals (95% CI) for UmA-pH < 7.20, < 7.10, and < 7.00, respectively, with Q1 as the reference. In Model 1, we calculated OR in univariate analysis. In Model 2, we calculated aOR adjusted for maternal age, BMI before pregnancy, and smoking status. For the vaginal delivery group, duration of labor was added in Model 1, which was then defined as Model 3. In this analysis, maternal age 20–29 years and BMI 18.5–25.0 kg/m^2^ were used as reference parameters. SPSS version 26 (IBM Corp., Armonk, NY, USA) was used for the statistical analysis. A *p*-value < 0.05 indicated statistical significance.

## 3. Result

**Figure 1:**
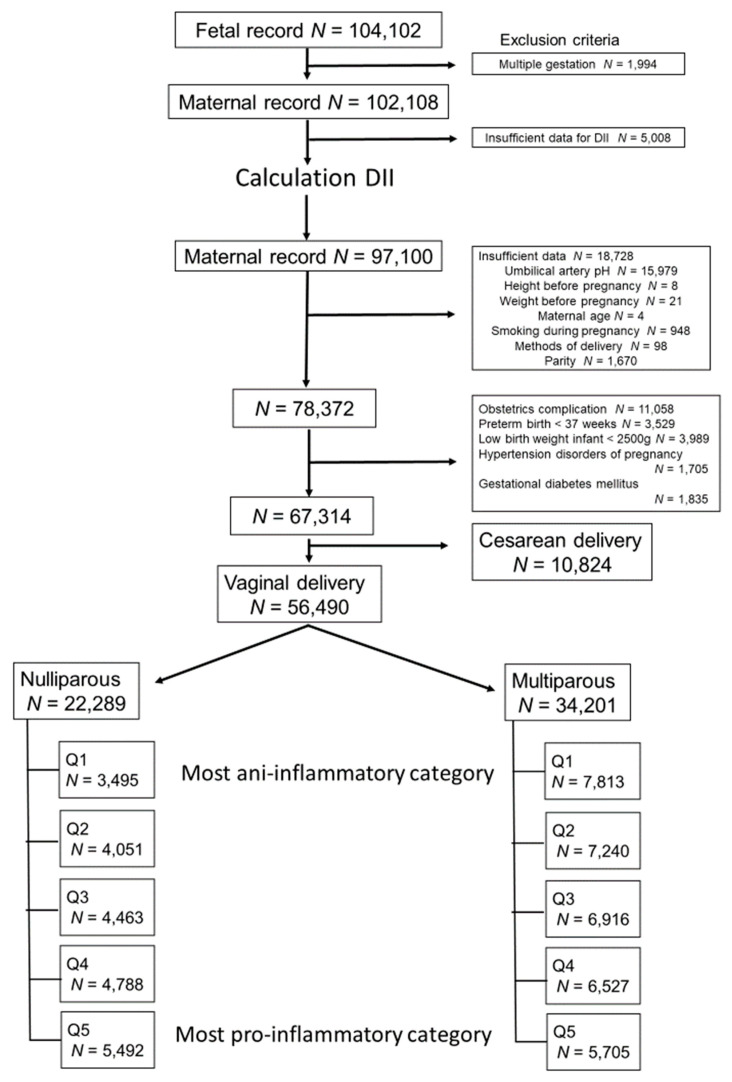
Study inclusion and exclusion criteria. DII: dietary inflammatory index.

**Figure 2:**
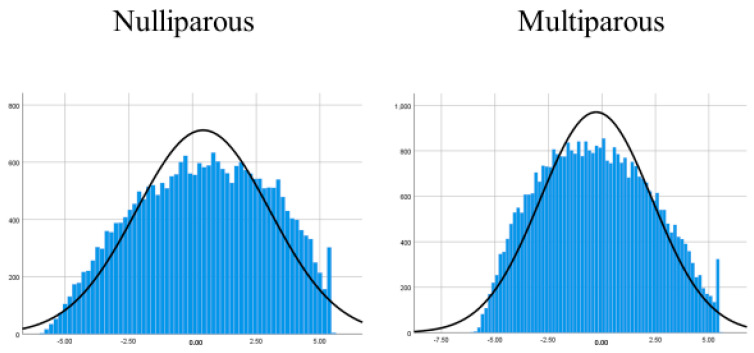
The frequency distribution of dietary inflammatory index (DII) score for two subgroups. The horizontal axis indicates DII score, and the vertical axis indicates the number of participants.

The total number of fetal records of infants delivered between 2011 to 2014 included in the JECS was 104,102. Of these, 1994 and 5008 infants were excluded because of multiple gestation and insufficient data for DII, respectively. Then, 18,728 and 11,058 participants were excluded because of insufficient data and obstetrics complication (3529 for PTB, 3989 for LBW, 1705 for HDP, and 1835 for GDM), respectively. Finally, 10,824 cases of cesarean delivery were excluded, and 56,490 participants were deemed eligible for this study and categorized into two groups on the basis of the method of delivery and parity. Finally, the groups were divided into five categories according to quintiles of their DII score (Figure 1). The median DII score ranged from −6.06 to +5.57 (mean: −0.04. standard deviation (±SD): 2.63) (Figure 2).

### 3.1. Maternal Background and Obstetric Outcomes

Table 1 summarizes the maternal background and obstetric outcomes according to quartiles for DII score. There was a significant difference in maternal age (both continuous value and category), BMI category before pregnancy, and smoking during pregnancy. While maternal age < 20 years was the most common in the highest DII score quartile (the most pro-inflammatory group), maternal age ≥ 30 years was the most common in the lowest DII score quartile (the most anti-inflammatory group). Both BMI < 18.5 and ≥ 25.0 were the highest in Q5 group, which is the highest pro-inflammatory group. Regarding the obstetric outcome, significant differences in the occurrence of UmA-pH < 7.20 were seen (*p* < 0.01). These occurrences were the highest among the most pro-inflammatory groups (7.1%). 

### 3.2. Comparison of DII and Obstetrics Characteristics among Subgroups

Table 2 shows the results of DII and the comparison of outcomes between nulliparous and multiparous women. A higher mean DII and the highest number in the Q5 group were observed among nulliparous women than among multiparous women. The occurrence of UmA-pH < 7.20 and < 7.10 was significantly higher in nulliparous women (both *p* < 0.01). The duration of labor was significantly longer in nulliparous women (9 h vs 4 h, *p* < 0.01).

### 3.3. Relationship between Dietary Inflammatory Index and Fetal Acidosis

Table 3 summarizes the association between DII categories and the risk of pH < 7.20. Among nulliparous women, the occurrence of pH < 7.20 was 9.4% in Q5, which was the highest among the five groups. However, both Model 2 and Model 3 show that Q5 had no significant risk for pH < 7.20 (aOR: 1.12, 95% CI: 0.97–1.31 and aOR: 1.12, 95% CI: 0.96–1.30, respectively).

Table 4 summarizes the association between DII categories and the risk of pH < 7.10. For nulliparous women, the risk of pH < 7.10 was significantly increased in the Q5 group (aOR: 1.64, 95% CI: 1.12–2.39) in Model 2 after adjusting for confounding factors. In Model 3, the risk of pH < 7.10 was still increased after adjusting for duration of labor (aOR: 1.64, 95% CI: 1.11–2.42). For multiparous women, no significant association was observed between DII category and the occurrence of pH < 7.10 in both Model 2 and Model 3.

Table 5 summarizes the association between DII category and risk of pH < 7.00. There was no association between DII category and risk of pH < 7.00 for both nulliparous and multiparous women.

## 4. Discussion

Several studies have examined the association between DII and non-communicable diseases [11], and a pro-inflammatory diet is thought to be positively related to systematic inflammation. Using a birth cohort study, Sen et al. [12] reported that a pro-inflammatory diet during pregnancy was associated with maternal systematic inflammation and fetal growth restriction. Yang et al. [13] reported that a pro-inflammatory diet during pregnancy increases high-sensitive C-reactive protein levels and neonatal birth weight. Ishibashi et al. [20] reported that a pre-pregnancy pro-inflammatory diet was associated with maternal leukocytosis in the first trimester, increased the risk of PTB < 34 weeks (aOR: 1.29, 95% CI 1.07–1.55), and < 2500 g LBW (aOR: 1.08, 95% CI 1.01–1.16), using the same birth cohort data set as the present study. This is the first report to examine the association between maternal diet during pregnancy and fetal acidosis, which was defined by a low cord pH at birth, using the largest birth cohort study in Japan. Our study suggested that vaginal delivery at first birth increased the risk of fetal pH < 7.10 among women who followed a high pro-inflammatory diet during pregnancy. Our results also suggested that the risk of fetal acidosis was not modified by the duration of labor among women at first birth.

In clinical practice, fetal oxygenation during labor is assessed on the basis of the fetal heart rate (FHR) pattern, using cardiotocography (CTG), which was introduced in the late 1960s, and it remains the gold standard for the management for intrapartum fetal assessment [28]. A severely low oxygenated status in the last half hour before delivery is associated with an increased risk of neonatal encephalopathy, cerebral palsy, and neonatal acidosis; thus, prompt delivery based on the severity of the abnormal FHR pattern and the progress of labor is recommended [28]. In Japan, as in other countries, a significant abnormal FHR pattern is an indication for CS, and the Japan Society of Obstetrics and Gynecology and Japan Association of Obstetricians and Gynecologists recommend that service providers pay high attention to the intrapartum FHR pattern for early medical intervention [29] and allow vaginal delivery only for the women who are considered to have a benign fetal condition on the basis of FHR pattern. Therefore, we excluded cases of cesarean delivery because these cases not only included selective cesarean delivery due to conditions such as malpresentation, previous history of cesarean delivery, or uterine surgery but also included emergent cesarean sections for potential fetal asphyxia, as in PTB, LBW, HDP, intrapartum significant abnormal FHR pattern, or labor dystocia. Consequently, this study is considered to have included cases with good intrapartum fetal oxygenation.

The study also showed that the risk of acidosis for the high DII group varied based on parity. The possible reason why a high DII increased the risk of fetal acidosis at birth only among nulliparous women is their duration of labor. Nulliparous women tend to have difficulty in the progress of labor. As shown in Table 2, the median duration of labor in nulliparous women was twice longer than that in multiparous women. Prolonged duration of labor is associated with an increased risk of fetal acidosis [30]. However, our study showed that a high DII diet during pregnancy still increased the risk of fetal acidosis with pH < 7.10 among nulliparous women after accounting for the duration of labor in Model 3 of the multivariate analysis, indicating that this risk does not only depend on the duration of labor.

Another possible reason, although very speculative, why high DII is associated with an increased risk for fetal acidosis is that maternal systemic inflammation, caused by a high DII diet, can progress to local organ inflammation, such as chorioamnionitis, so called intrauterine inflammation. Intrauterine inflammation with an intrapartum hypoxic condition has a synergic effect on the fetal deteriorated condition [31]. Nevertheless, an intrapartum FHR pattern in cases with intrauterine inflammation is not well understood. In humans, several observational studies have reported that intrauterine inflammation could cause an abnormal FHR pattern [32,33,34,35]. Salafia et al. [36] reported that the sensitization of the smooth muscle of the umbilical vessel by the surrounding inflammatory cytokines may cause uterine contractions or other minor trauma, as well as a potentially more severe abnormal FHR pattern. However, human observational studies are limited to analyses of FHR patterns that are mostly benign, as it is not ethical to observe FHR patterns in those who are potentially at an increased risk of fetal acidosis without intervention. However, in an experiment involving pregnant sheep, some underlying conditions, such as a fetal acute hypoxic state due to magnesium sulfate use, were found to modify the FHR pattern. This could result in underestimation of the actual fetal condition, causing a delay in the management of the abnormal FHR pattern during labor [37]. Kyozuka et al. [38] reported that development of severe acidosis in a fetus because of lipopolysaccharide induced intrauterine inflammation with an impaired FHR pattern indicates low oxygenation. Therefore, a scenario in which a high DII during pregnancy can increase the risk of fetal acidosis at birth is when the intrapartum FHR pattern is modified because of severe intrauterine inflammation with resultant delay in obstetric intervention on the basis of the FHR pattern.

This is the first-large-scale study conducted in Japan by the Japanese government with meticulous attention to data collection. Therefore, this study is considered representative of the general pregnant population in Japan [39]. Additionally, we stratified participants according to the mode of delivery and parity, which affected the oxidative conditions at birth, with a sufficiently large number of participants in each group. Nevertheless, this study also has potential limitations that should be considered. First, although we had previously reported that a high DII diet before pregnancy is associated with leukocytosis in the first trimester, we did not measure plasma inflammatory markers, such as white blood cell, cytokines, such as CRP, IL-6, and TNFα, at the time of delivery in the present study. We were also not aware of the histological findings of the inflamed placenta or umbilical cord in each case. Second, we had no records on FHR monitoring in cases of acidosis, which has broad acceptance as a valuable aid in optimizing fetal outcomes. Therefore, we are not aware of the FHR patterns of cases of severe acidosis due to a high DII among nulliparous women. Third, although we accounted for some confounding factors and excluded cases with obstetric complications, unknown factor that affect the fetal pH condition might exist.

## 5. Conclusions

In this study, we found that a pro-inflammatory diet during pregnancy was a risk factor for fetal acidosis at birth among nulliparous women with vaginal delivery. Furthermore, these risks were not modified by the duration of labor. Conventionally, obstetric clinical practice has focused on perinatal asphyxia solely by fetal oxygenation just before delivery. Our study indicated that dietary habits may affect intrapartum fetal oxygenation via modification of the intrapartum FHR pattern. The JECS is expected to continue to examine the impact of several environmental factors on the long-term children’s outcomes. Thus, we hope to further investigate the influence of a high DII during pregnancy on short- and long-term neurodevelopment outcomes.

## Figures and Tables

**Table 1:** Maternal medical background and obstetric outcomes.

Variable	Q1 (Most Anti-Inflammatory Group)	Q2	Q3	Q4	Q5 (Most Pro-Inflammatory Group)	*p*-Value
	*n* = 11,308	*n* = 11,291	*n* = 11,379	*n* = 11,315	*n* = 11,197	
Maternal background						
DII, mean (±SD)	−3.67 (0.79)	−1.64 (0.49)	−0.02 (0.46)	1.62 (0.50)	3.72 (0.81)	<0.01 ^a^
Maternal age, mean year (±SD)	31.9 (4.7)	31.4 (4.7)	31.0 (4.8)	30.5 (4.9)	29.3 (5.2)	<0.01 ^a^
Maternal age category, %						
≤19	0.4	0.5	0.7	0.9	1.9	<0.01 ^b^
20–29	30.5	34.2	37.9	42.0	50.5	
≥30	69.1	65.3	61.4	57.1	47.6	
BMI, %						
<18.5	15.7	16.2	16.8	16.7	16.9	<0.01 ^b^
18.5–24.9	76.0	75.9	74.8	74.4	72.8	
≥25.0	8.3	7.9	8.4	8.9	10.3	
Primipara, %	30.9	35.9	39.2	42.3	49.0	<0.01 ^b^
Smoking, %	3.4	3.3	3.6	4.9	6.3	<0.01 ^b^
UmA-pH, mean (±SD)	7.31 (0.14)	7.32 (0.08)	7.31 (0.10)	7.31 (0.10)	7.31 (0.15)	<0.01 ^a^
UmA-pH < 7.20, %	6.1	6.3	6.6	5.9	7.1	<0.01 ^b^
UmA-pH < 7.10, %	1.0	1.0	1.0	1.1	1.2	0.24 ^b^
UmA-pH < 7.00, %	0.2	0.2	0.2	0.2	0.3	0.39 ^b^

DII: dietary inflammatory index, SD: standard deviation, BMI: body mass index, UmA: Umbilical Artery. ^a^ *p*-value, one-way analysis of variance. ^b^ *p*-value, chi-square test.

**Table 2:** Comparison of DII and obstetrics characteristics among subgroups.

	Nulliparous	Multiparous	*p*-Value
	*n* = 22,289	*n* = 34,210	
Variable			
Maternal age, mean year (±SD)	29.3 (5.1)	31.8 (4.6)	<0.01 ^a^
DII, mean (±SD)	0.41 (2.61)	−0.27 (2.61)	<0.01 ^a^
DII, %			
Q1(Most anti-inflammatory)	15.8	22.8	<0.01 ^b^
Q2	18.0	21.2	
Q3	19.9	20.1	
Q4	21.5	19.2	
Q5 (Most pro-inflammatory)	24.9	16.8	
UmA-pH, mean (±SD)	7.30 (0.11)	7.32 (0.12)	<0.01 ^a^
UmA-pH < 7.20, %	8.7	4.9	<0.01 ^b^
UmA-pH < 7.10, %	1.4	0.8	<0.01 ^b^
UmA-pH < 7.00, %	0.2	0.2	0.18 ^b^
Duration of labor, median hours (IQR)	9 (6–15)	4 (3–7)	<0.01 ^c^

DII: dietary inflammatory index, SD: standard deviation, PTB: Preterm birth, LBW: Low birth weight, UmA: Umbilical Artery, IQR: Interquartile range; ^a^ *p*-value, *t*-test. ^b^ *p*-value, chi-square test., ^c^ *p*-value, Mann–Whitney *U* test.

**Table 3:** Relationship between dietary inflammatory index and umbilical artery pH < 7.20.

Parity		Q1(Most Anti-Inflammatory Group)	Q2	Q3	Q4	Q5 (Most Pro-Inflammatory Group)
Nulliparous						
	Number	3495	4051	4463	4788	5492
	Case, %	8.5	8.9	8.7	7.9	9.4
	Model 1 OR (95% CI)	Ref	1.05 (0.90–1.23)	1.03 (0.88–1.20)	0.92 (0.79–1.08)	1.12 (0.96–1.30)
	Model 2 aOR (95% CI)	Ref	1.04 (0.89–1.22)	1.03 (0.88–1.21)	0.93 (0.79–1.09)	1.12 (0.97–1.31)
	Model 3 aOR (95% CI)	Ref	1.04 (0.90–1.22)	1.03 (0.88–1.21)	0.94 (0.80–1.10)	1.12 (0.96–1.30)
Multiparous						
	Number	7813	7240	6916	6527	5705
	Case, %	5.1	4.9	5.1	4.5	4.8
	Model 1 OR (95% CI)	Ref	0.96 (0.83–1.11)	1.02 (0.88–1.18)	0.88 (0.75–1.02)	0.94 (0.80–1.10)
	Model 2 aOR (95% CI)	Ref	0.96 (0.83–1.11)	1.03 (0.89–1.19)	0.89 (0.76–1.04)	0.97 (0.83–1.14)
	Model 3 aOR (95% CI)	Ref	0.96 (0.83–1.11)	1.02 (0.88–1.19)	0.88 (0.75–1.03)	0.97 (0.83–1.14)

OR: odds ratio, aOR: adjusted odds ratio, CI: confidence interval, Ref: reference. In Model 2, aOR was calculated by logistic regression analysis, using maternal age, body mass index before pregnancy, and maternal smoking status. In Model 3, aOR was calculated by Model 2 and duration of labor.

**Table 4:** Relationship between dietary inflammatory index and umbilical artery pH < 7.10.

Parity		Q1(Most Anti-Inflammatory Group)	Q2	Q3	Q4	Q5 (Most Pro-Inflammatory Group)
Nulliparous						
	Number	3495	4051	4463	4788	5492
	Case, %	1.1	1.5	1.3	1.4	1.8
	Model 1 OR (95% CI)	Ref	1.33 (0.89–2.00)	1.19 (0.79–1.78)	1.26 (0.85–1.87)	1.61 (1.11–2.34)
	Model 2 aOR (95% CI)	Ref	1.31 (0.87–1.97)	1.12 (0.78–1.77)	1.28 (0.86–1.90)	1.64 (1.12–2.39)
	Model 3 aOR (95% CI)	Ref	1.33 (0.88–2.02)	1.22 (0.80–1.84)	1.32 (0.88–1.98)	1.64 (1.11–2.42)
Multiparous						
	Number	7813	7240	6916	6527	5705
	Case, %	0.9	0.7	0.7	0.9	0.7
	Model 1 OR (95% CI)	Ref	0.83 (0.58–1.19)	0.82 (0.57–1.18)	0.97 (0.69–1.39)	0.78 (0.53–1.15)
	Model 2 aOR (95% CI)	Ref	0.84 (0.59–1.20)	0.84 (0.59–1.21)	1.00 (0.70–1.42)	0.84 (0.57–1.24)
	Model 3 aOR (95% CI)	Ref	0.82 (0.57–1.17)	0.79 (0.55–1.14)	0.95 (0.67–1.36)	0.81 (0.55–1.21)

OR: odds ratio, aOR: adjusted odds ratio, CI: confidence interval, Ref: reference. In Model 2, aOR was calculated by logistic regression analysis, using maternal age, BMI before pregnancy, and maternal smoking status. In Model 3, aOR was calculated by Model 2 and duration of labor.

**Table 5:** Relationship between dietary inflammatory index and umbilical artery pH < 7.00.

Parity		Q1(Most Anti-Inflammatory Group)	Q2	Q3	Q4	Q5 (Most Pro-Inflammatory Group)
Nulliparous						
	Number	3495	4051	4463	4788	5492
	Case, %	0.1	0.3	0.1	0.2	0.3
	Model 1 OR (95% CI)	Ref	2.59 (0.84–8.05)	0.98 (0.26–3.65)	1.64 (0.51–5.34)	2.55 (0.85–7.63)
	Model 2 aOR (95% CI)	Ref	2.67 (0.86–8.30)	1.05 (0.28–3.92)	1.79 (0.55–5.83)	2.26 (0.87–8.21)
	Model 3 aOR (95% CI)	Ref	2.43 (0.77–7.63)	1.05 (0.28–3.90)	1.78 (0.55–5.79)	2.47 (0.80–7.64)
Multiparous						
	Number	7813	7240	6916	6527	5705
	Case, %	0.2	0.1	0.2	0.1	0.2
	Model 1 OR (95% CI)	Ref	0.75 (0.32–1.75)	1.04 (0.48–2.23)	0.74 (0.31–1.78)	1.16 (0.52–2.59)
	Model 2 aOR (95% CI)	Ref	0.76 (0.32–1.78)	1.06 (0.49–2.33)	0.76 (0.31–1.84)	1.22 (0.54–2.74)
	Model 3 aOR (95% CI)	Ref	0.70 (0.28–1.62)	0.97 (0.44–2.18)	0.76 (0.31–1.83)	1.22 (0.55–2.75)

OR: odds ratio, aOR: adjusted odds ratio, CI: confidence interval, Ref: reference. In Model 2, aOR was calculated by logistic regression analysis, using maternal age, body mass index before pregnancy, and maternal smoking status. In Model 3, aOR was calculated by Model 2 and duration of labor.
